# Childhood maltreatment history and attention bias variability in healthy adult women: role of inflammation and the *BDNF* Val66Met genotype

**DOI:** 10.1038/s41398-021-01247-4

**Published:** 2021-02-11

**Authors:** Hiroaki Hori, Mariko Itoh, Mingming Lin, Fuyuko Yoshida, Madoka Niwa, Yuko Hakamata, Mie Matsui, Hiroshi Kunugi, Yoshiharu Kim

**Affiliations:** 1 Department of Behavioral Medicine, National Institute of Mental Health, National Center of Neurology and Psychiatry, Tokyo, Japan; 2 Center for Environmental and Health Sciences, Hokkaido University, Sapporo, Japan; 3 Department of Mental Disorder Research, National Institute of Neuroscience, National Center of Neurology and Psychiatry, Tokyo, Japan; 4 Department of Clinical Psychology, International University of Health and Welfare, Tokyo, Japan; 5 Department of Clinical Cognitive Neuroscience, Institute of Liberal Arts and Science, Kanazawa University, Kanazawa, Japan; 6 Department of Psychiatry, Teikyo University School of Medicine, Tokyo, Japan

**Keywords:** Human behaviour, Clinical genetics, Psychiatric disorders, Diagnostic markers

## Abstract

Childhood maltreatment has been associated with greater attention bias to emotional information, but the findings are controversial. Recently, a novel index of attention bias, i.e., attention bias variability (ABV), has been developed to better capture trauma-related attentional dysfunction. However, ABV in relation to childhood trauma has not been studied. Here, we examined the association of childhood maltreatment history with attention bias/ABV in 128 healthy adult women. Different types of childhood maltreatment were assessed with the Childhood Trauma Questionnaire. Attention bias/ABV was measured by the dot-probe task. Possible mechanisms whereby childhood maltreatment affects attention bias/ABV were also explored, focusing on blood proinflammatory markers and the *BDNF* Val66Met polymorphism. We observed a significant positive correlation between childhood emotional abuse and ABV (*P* = 0.002). Serum high-sensitivity tumor necrosis factor-α levels were significantly positively correlated with ABV (*P* < 0.001), but not with childhood maltreatment. Jonckheere–Terpstra trend test showed a significant tendency toward greater ABV with increasing numbers of the *BDNF* Met alleles (*P* = 0.021). A two-way analysis of variance further revealed that the genotype-by-emotional abuse interaction for ABV was significant (*P* = 0.022); individuals with the Val/Met and Met/Met genotypes exhibited even greater ABV when childhood emotional abuse was present. These results indicate that childhood emotional abuse can have a long-term negative impact on emotional attention control. Increased inflammation may be involved in the mechanism of ABV, possibly independently of childhood maltreatment. The *BDNF* Met allele may dose-dependently increase ABV by interacting with childhood emotional abuse.

## Introduction

Childhood maltreatment, a form of early-life stress, is a major public health concern that has devastating consequences for both the affected individuals and society as a whole^1^. Accumulated evidence indicates that childhood maltreatment, including emotional/physical abuse, emotional/physical neglect, and sexual abuse, can significantly increase the risk of developing mental disorders in later life, which include—but not limited to—depression and posttraumatic stress disorder (PTSD)^2–4^. Although the mechanism by which childhood maltreatment leads to the development of these disorders remains unclear, it has been postulated that biased attention to emotional stimuli is involved in this process^5,6^.

Attentional processing of emotional information is essential for emotion regulation, yet this is not always a straightforward process. Attention bias, a type of cognitive bias, is the tendency to pay more attention to emotionally salient stimuli than to neutral ones. Attention bias has been shown in individuals with a history of childhood maltreatment^7–10^, and this bias is considered as one of the most significant cognitive features in individuals exposed to early trauma^5,6^. Attention bias has also been observed in a variety of psychiatric conditions, including anxiety^11^, depression^12^, and PTSD^13^.

One of the most widely used experimental paradigms for measuring attention bias is the dot-probe task. Using this task, several studies have investigated threat-related attention bias in relation to childhood maltreatment. However, their findings have been mixed, such that some studies report a bias toward threat^9,14^ whereas others describe no bias^15^ or even a bias away from threat^16–18^. Similar inconsistencies exist in the PTSD literature; some studies report a bias toward threat^13,19^, others no bias^20,21^, and still others a bias away from threat^22–24^.

More recently, a novel index of attention bias, i.e., attention bias variability (ABV), has been used to better capture trauma-related attentional dysfunction^25,26^. Unlike the unidirectional assessment of attention bias, ABV reflects dynamic fluctuations in attention both toward and away from emotional information. It is also pointed out that such attention bias toward and away from threatening stimuli can reflect two main symptoms of PTSD, namely hypervigilance and avoidance, respectively^20,27^. In accordance with this, studies have demonstrated that PTSD patients exhibit greater ABV than control subjects and that ABV positively correlates with PTSD severity^20,21,25,28,29^. A randomized controlled trial comparing the efficacy of attention bias modification and attention control training for PTSD showed that the latter, but not the former, significantly reduced ABV and that reductions in ABV partially mediated improvement in PTSD symptoms^28^. This suggests that ABV is not merely an epiphenomenon of PTSD but can contribute to initiate and maintain this disorder. To our knowledge, however, no studies have examined the association of childhood maltreatment history with ABV.

Various lines of research have investigated biological mechanisms underlying the link between early trauma and long-term alterations in cognitive styles and psychopathology that persist into adulthood^30,31^. One of these mechanisms is suggested to be dysregulation of the immune and inflammatory system^32,33^. This has been supported by the evidence that childhood maltreatment leads to increased inflammation as indexed by peripheral proinflammatory markers^34,35^ and that inflammation is associated with emotional attention^36,37^ and with psychiatric disorders including depression and PTSD^38–40^. While several studies have investigated the association between inflammation (e.g., inflammatory stress and circulating proinflammatory markers) and attention bias^37,41^, we are not aware of any studies that examined the association between inflammation and ABV.

It is well-known that genetic factors and their complex interactions with environmental factors can increase the risk for developing multifactorial disorders, such as depression and PTSD^42,43^. Of the candidate genes, the brain-derived neurotrophic factor (*BDNF*) gene is implicated in both depression^44,45^ and PTSD^46–48^. The *BDNF* gene encodes a neurotrophin that is involved in neuronal growth, differentiation, maturation, and survival in immature neurons and in synaptic plasticity, neurotransmission, and receptor sensitivity in mature neurons^49^. *BDNF* has a functional single-nucleotide polymorphism (SNP) known as Val66Met, which is shown to reduce the activity-dependent secretion of BDNF^50^. Studies have demonstrated that this SNP interacts with childhood adversity to affect cognition, emotion, and psychopathology^51–55^; for example, it interacts with childhood maltreatment to influence the development and severity of depression^52,53^ and PTSD^54^. This SNP has also been associated with attention bias^56,57^ and circulating inflammatory markers^58,59^. Furthermore, this SNP has been associated with exaggerated expression of memories with negative emotional valence in both mice^60^ and human PTSD patients^61^. These findings together suggest that the *BDNF* Val66Met polymorphism may affect attention bias and ABV by interacting with childhood maltreatment.

In this study, we investigated the association of childhood maltreatment history with attention bias and ABV in healthy adult women. In doing so, we distinguished different maltreatment types, namely emotional abuse, physical abuse, sexual abuse, emotional neglect, and physical neglect, given that long-term effects on cognition and emotion can differ across these maltreatment types. We further explored the possible mechanism by which childhood maltreatment affects attention bias/ABV, focusing on blood proinflammatory markers and the *BDNF* Val66Met genotype.

## Materials and methods

### Participants

A total of 128 healthy women (age range: 20–64 years) participated in this study. This sample size was determined by referring to previous studies on attention bias in relation to childhood maltreatment^5,8,10^. All participants were native Japanese speakers, almost all of whom resided in the western part of the Tokyo metropolitan. They were recruited from the community through advertisements in free local magazines, our website, and university campuses, and by word of mouth. The validated Japanese version^62^ of the Mini International Neuropsychiatric Interview (M.I.N.I.)^63^ was administered to the participants in order to confirm the absence of any current Axis-I psychiatric disorders. In addition, the validated Japanese version^64^ of the Posttraumatic Diagnostic Scale (PDS)^65^ was used to further ascertain the absence of PTSD diagnosis. Additional exclusion criteria were: current severe physical illness or apparent intellectual disability, past/current contact to psychiatric services, and past/current use of psychotropic medications.

This study was approved by the ethics committee of the National Center of Neurology and Psychiatry, Japan, and was conducted in accordance with the Declaration of Helsinki. Written informed consent was obtained from all participants after the study procedures had been fully explained.

### Questionnaires

Detailed information is provided in Supplementary Methods.

#### The Posttraumatic Diagnostic Scale (PDS)^65^

The PDS was created in accordance with the diagnostic criteria of DSM-IV PTSD^65^. It consists of four parts that assess traumatic experiences, PTSD symptoms during the past month, and the associated functional impairments; when the first part confirms the absence of traumatic experiences, no further assessment of symptomatology is made.

#### The Childhood Trauma Questionnaire (CTQ)^66^

The CTQ is widely used to assess the history of childhood maltreatment^66^. The commonly used 28-item version of CTQ includes 25 clinical items and 3 validity items. The 25 items load onto 5 subscales that assess different types of childhood maltreatment, including emotional abuse, physical abuse, sexual abuse, emotional neglect, and physical neglect. All items are rated on a 5-point scale ranging from 1 to 5, with higher scores indicating more severe maltreatment. Cutoff scores for each subscale are defined in the manual of the CTQ^67^; the cutoff scores distinguishing between “none”/“low” for emotional abuse, physical abuse, sexual abuse, emotional neglect, and physical neglect are 8/9, 7/8, 5/6, 9/10, and 7/8, respectively. Cronbach α coefficients of the five CTQ subscales, i.e., emotional abuse, physical abuse, sexual abuse, emotional neglect, and physical neglect, in the present sample, were 0.84, 0.54, 0.56, 0.89, and 0.27, respectively.

#### The Beck Depression Inventory-II (BDI-II)^68^

Depressive symptoms were assessed by the validated Japanese version^69^ of the BDI-II, a 21-item self-report questionnaire for depression severity during the past 2 weeks. Each item is scored on a 4-point scale from 0 to 3, with higher scores indicating greater depressive symptoms. The cutoff BDI-II total score distinguishing between “minimal”/“mild” depression is 13/14^68^. Cronbach α coefficient of the BDI-II in this sample was 0.84.

#### The State-Trait Anxiety Inventory (STAI)^70^

Anxiety symptoms were assessed by the validated Japanese version^71^ of the STAI, a self-report questionnaire widely used to assess anxiety. It consists of two subscales for the state (STAI-S) and trait (STAI-T) anxiety, both comprising 20 items that are scored on a 4-point scale from 1 to 4; higher scores indicate greater anxiety. Cronbach α coefficients of the STAI-S and STAI-T in this sample were 0.77 and 0.60, respectively.

### Cognitive measures

#### Attention bias and ABV

The dot-probe task was used to measure attention bias and ABV.

Before each trial, a white fixation cross (“+”) appeared in the center of the black display for 500 ms. Pairs of words were then presented for 1000 ms, one on top of the other. Immediately after their presentation, a probe (“←” or “→”) appeared in a location that corresponded to the center of one of the two words. Depending on the direction of the arrow, participants were instructed to press either a left or right key with the index or ring finger as quickly as possible. The two fingers were positioned above the two keys throughout the task. The arrow disappeared with the correct keypress. The next trial started after an interval of 500 ms. Six practice trials contained pairs of emotionally neutral words that were not displayed again. In the experimental 224 trials, there were 152 trials with pairs of generally negative words (e.g., “imprisonment”, “thief”, etc.) and neutral ones (e.g., “wheat”, “product”, etc.), and 72 trials with two neutral words. Types of trials were randomly interleaved. The position of the negative and neutral words and the position of the probe arrow were counterbalanced. To calculate attention bias, trials with the negative and neutral pairs were analyzed. After trials with errors and those with unnatural reaction times (RTs) (defined as >2000 ms or <150 ms) were removed, mean RTs were calculated separately for probes (a) replacing the same position as negative word (i.e., “congruent condition”) and (b) replacing the other position from negative word (“incongruent condition”). Trials above 2 standard deviations (SD) of the participant’s mean for each probe condition were excluded from further analyses; in the total participants, 5.5% and 5.8% of all trials for the congruent and incongruent conditions (respectively) were excluded.

An attention bias score for negative words was calculated as follows:

“Attention bias score” = “RTs for incongruent condition” − “RTs for congruent condition”.

Positive values reflect attention toward the negative words, and negative values reflect attention away from the negative words.

To further calculate ABV, all trials were split into eight sequential bins, and attention bias scores were calculated for each bin^20^. The SD of attention bias scores across bins was calculated and divided by mean RT to correct for variance in RTs^72,73^. Thus, ABV was calculated using the following equation, with greater values reflecting the instability of attention bias:

$${\mathrm{SD}}_{{\mathrm{AB}}} = \sqrt {\frac{{\mathop {\sum }\nolimits_{i = 1}^8 \left( {{\mathrm{AB}}_i - \overline {{\mathrm{AB}}} } \right)^2}}{{{\mathrm{n}} - 1}}}$$

$${\mathrm{ABV}} = \frac{{{\mathrm{SD}}_{{\mathrm{AB}}}}}{{\overline {{\mathrm{RT}}} }}$$

where

*i* indicates the bin number,

*n* indicates the total number of bins (i.e., “8”),

AB indicates attention bias scores,

and RT indicates reaction time.

#### Attention ability and global cognitive function

We also examined general attention ability, as it can affect attention bias/ABV^74^. For this purpose, the validated Japanese version^75^ of the Repeatable Battery for the Assessment of Neuropsychological Status (RBANS)^76^ was used. While the full version of RBANS was administered to all participants, our analyses focused on the two cognitive indices, namely attention and total score. Age-corrected standardized scores, with a population mean of 100 and SD of 15, are calculated for each cognitive domain. Additional details are provided in Supplementary Methods.

### Measurement of proinflammatory markers

Of the total 128 participants who completed the psychological and cognitive assessments, 118 individuals also participated in the blood testing to examine proinflammatory markers, including high-sensitivity tumor necrosis factor-α (hsTNF-α), interleukin-6 (IL-6), and high-sensitivity C-reactive protein (hsCRP). Reasons for the attrition of ten participants were: informed consent to the blood testing was not given, and blood sampling was technically difficult. This blood sampling was performed on the same day as the psychological/cognitive assessments. The samples were collected from each participant around noon (before lunch), between 11:30 AM and 12:30 PM. Levels of hsTNF-α, IL-6, and hsCRP were measured at a clinical laboratory (SRL Inc., Tokyo, Japan). Serum hsTNF-α, IL-6, and hsCRP levels were measured by enzyme-linked immunosorbent assay, chemiluminescent enzyme immunoassay, and nephelometry, respectively. There were no participants who showed hsCRP levels >10,000 ng/ml (i.e., 10 mg/l), an objective feature of acute infection^77^. Additional details are described in Supplementary Methods.

#### *BDNF* Val66Met genotyping

Of the total 128 participants, 107 also participated in the genetic testing by blood sampling. This sample attrition was due to the fact that several participants did not provide informed consent to genetic testing, in addition to the reasons related to blood sampling as described in the previous section.

Genomic DNA was prepared from venous blood according to standard procedures. Rs6265 (Val66Met) was genotyped using the TaqMan SNP Genotyping Assays (assay ID: C__11592758_10). The thermal cycling conditions for polymerase chain reaction were: 1 cycle at 95 °C for 10 min followed by 45 cycles of 95 °C for 15 s and 60 °C for 1 min. The allele-specific fluorescence was measured using ABI PRISM 7900 Sequence Detection Systems (Applied Biosystems, Foster City, CA). All samples had a genotyping call rate of 97% or greater.

### Statistical analysis

Averages are reported as “mean ± SD”, or “median (interquartile range)” where appropriate. The Kolmogorov–Smirnov normality test showed that ABV satisfied the assumption of the normal distribution while attention bias, CTQ scores, and inflammatory marker levels did not.

Correlations among CTQ scores, attention ability/bias indices, and inflammatory markers were examined using Spearman’s rank-order correlation, considering that most of these data deviated from the normal distribution.

The relationship of the *BDNF* Val66Met polymorphism with attention bias indices was examined using the Jonckheere–Terpstra trend test, a rank-based nonparametric test for determining if there is a statistically significant trend between two variables. In this analysis, the three genotype groups (i.e., Val/Val vs. Val/Met vs. Met/Met) were distinguished, and the dose–response relationship between the number of Met alleles and attention bias index was examined. When there was a significant trend between the genotype groups and attention bias index, a two-way analysis of variance (ANOVA) was used to further examine the interaction effect of genotype (i.e., Val/Val vs. Val/Met vs. Met/Met) and childhood maltreatment (i.e., presence vs. absence based on the CTQ cutoff score) on the attention bias index.

In addition, a mediation analysis was used to explore the potential mediation of depressive symptoms that might underlie the relationship between childhood maltreatment and ABV; this analysis was performed as a post hoc exploratory analysis. It was conducted with the Mplus version 7^78^, using the following command:

VARIABLE: NAMES = X Y M; USEVARIABLES = X Y M;

ANALYSIS: TYPE = GENERAL; ESTIMATOR = ML;

MODEL: Y ON M X; M ON X;

MODEL INDIRECT: Y IND M X;

OUTPUT: STANDARDIZED(STDYX);

Statistical significance was set at two-tailed *P* < 0.05 unless otherwise specified; while Bonferroni-corrected *P* values were applied to the five CTQ domains (i.e., *P* < 0.01) and three inflammatory markers (i.e., *P* < 0.016) in order to adjust for the multiple testing. All statistical analyses, except for the mediation analysis, were performed using the Statistical Package for the Social Sciences version 25 (IBM Corp., Tokyo, Japan).

## Results

### Demographic and psychological characteristics

The demographic and psychological characteristics of the sample are shown in Table 1. Participants, all women, were on average mid-to-late thirties and tertiary educated, and predominantly nonsmokers. The median and interquartile range of the five CTQ domains indicated that while childhood emotional neglect was relatively frequently observed, emotional abuse and physical neglect were less frequent, and physical abuse and sexual abuse were totally absent in most participants (Table 1); of the 128 individuals, 111 (86.7%) reported no physical abuse and 115 (89.8%) reported no sexual abuse (i.e., scored “5”). The mean RBANS total score indicated that the majority of our participants were neuropsychologically normal.

**Table 1:** Demographic and psychological characteristics of the sample.

Variable	Total participants (*n* = 128)
Age, years: mean ± SD	36.4 ± 13.0
Education level^a^: median (IQR)	3.0 (3.0–4.0)
Smoking: yes, *n* (%)	20 (15.6)
BDI-II, total score: mean ± SD	5.8 ± 5.3
*STAI: mean* *±* *SD*	
State	37.2 ± 7.9
Trait	39.5 ± 8.7
*CTQ: median (IQR)*	
Emotional abuse	6.0 (5.0–9.0)
Physical abuse	5.0 (5.0–5.0)
Sexual abuse	5.0 (5.0–5.0)
Emotional neglect	11.0 (8.0–15.0)
Physical neglect	6.0 (5.0–8.0)
*RBANS: mean* *±* *SD*	
Total score	104.5 ± 14.2
Attention	105.9 ± 15.0
*Dot-probe task: median (IQR)*	
Attention bias	−0.77 (−8.2 to 5.1)
Attention bias variability	0.045 (0.032–0.058)

*BDI-II* Beck Depression Inventory-II, *STAI* State-Trait Anxiety Inventory, *CTQ* Childhood Trauma Questionnaire, *RBANS* Repeatable Battery for the Assessment of Neuropsychological Status, *SD* standard deviation, *IQR* interquartile range.

*Notes*: ^a^Coded as follows: 1, junior high school graduate; 2, high school graduate; 3, some college graduate/partial university; 4, university graduate; 5, graduate school graduate.

Neither age nor education was significantly correlated with CTQ 5 domains, attention bias, or ABV as indicated by Spearman’s rho (all *P* > 0.1). Smokers and nonsmokers did not significantly differ in CTQ 5 domains, attention bias, or ABV as examined by the Mann–Whitney *U* test (all *P* > 0.1).

### Correlations among attention indices and psychological variables

Average scores of the three attention indices, including attention ability (assessed with RBANS), attention bias (dot-probe task), and ABV (dot-probe task) are presented in Table 1.

Correlations of attention ability with attention bias and ABV were both at a trend level (rho = −0.150, *P* = 0.091 and rho = −0.153, *P* = 0.085, respectively). The correlation between attention bias and ABV was not significant (rho = −0.042, *P* = 0.638).

**Fig. 1:**
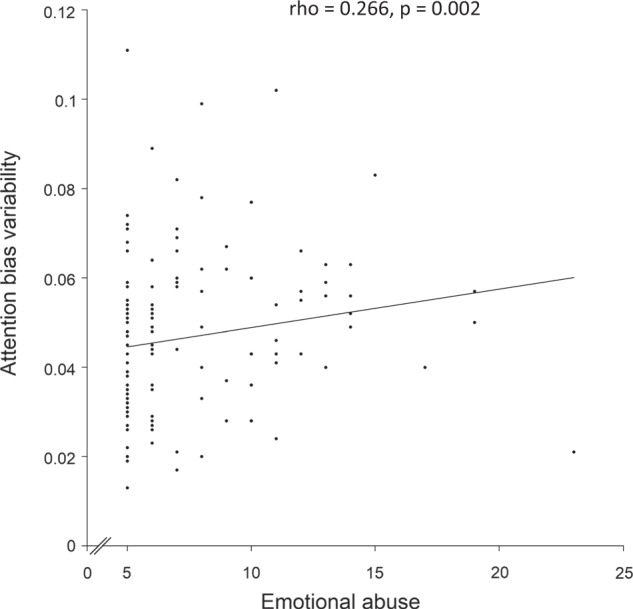
Scatterplot showing the relationship between childhood emotional abuse and attention bias variability. History of childhood emotional abuse was significantly positively correlated with attention bias variability (rho = 0.266, *P* = 0.002).

Correlations of the five CTQ subscales with the three attention indices are shown in Table 2. Emotional abuse was significantly positively correlated with ABV (rho = 0.266, *P* = 0.002) while the other types of maltreatment were not significantly correlated with any of the attention indices (all *P* > 0.1). The relationship between emotional abuse and ABV is plotted in Fig. 1.

**Table 2:** Correlations between childhood maltreatment and attention indices in total participants (*n* = 128).

	Attention ability (RBANS)	Attention bias (DPT)	Attention bias variability (DPT)
Emotional abuse (CTQ)	−0.061	−0.044	0.266^**^
Physical abuse (CTQ)	0.027	0.014	0.082
Sexual abuse (CTQ)	−0.007	−0.103	0.078
Emotional neglect (CTQ)	0.053	−0.044	0.040
Physical neglect (CTQ)	−0.104	−0.061	0.043

*CTQ* Childhood Trauma Questionnaire, *RBANS* Repeatable Battery for the Assessment of Neuropsychological Status, *DPT* dot-probe task.

*Notes*: ^**^P = 0.002. Correlations were calculated using Spearman’s ρ.

Regarding the relationships with depressive and anxiety symptoms, ABV was significantly correlated with the BDI-II total score (rho = 0.252, *P* = 0.004) but not with STAI-S or STAI-T scores (both *P* > 0.05); while attention ability and attention bias were not significantly correlated with BDI-II or STAI-S/-T scores (all *P* > 0.05).

### Relationships among childhood maltreatment, inflammation, and attention indices

Median (interquartile range) levels of hsTNF-α, IL-6, and hsCRP were 0.695 (0.528–0.805) pg/ml, 0.800 (0.600–1.100) pg/ml, and 198.5 (103.8–425.5) ng/ml, respectively.

None of the five CTQ subscales were significantly correlated with any of the three proinflammatory marker levels (all *P* > 0.1).

Correlations of the three proinflammatory marker levels with the three attention indices are shown in Table 3. hsTNF-α levels were significantly positively correlated with ABV (rho = 0.302, *P* < 0.001) while IL-6 and hsCRP levels were not significantly correlated with any of the attention indices (all *P* > 0.1). The relationship between hsTNF-α levels and ABV is plotted in Supplementary Fig. 1.

**Table 3:** Correlations between proinflammatory marker levels and attention indices in a subset of participants (*n* = 118).

	Attention ability (RBANS)	Attention bias (DPT)	Attention bias variability (DPT)
hsTNF-α	−0.140	0.009	0.302^***^
IL-6	0.101	0.021	0.002
hsCRP	0.054	−0.095	0.139

*hsTNF-α* high-sensitivity tumor necrosis factor-α, *IL-6* interleukin-6, *hsCRP* high-sensitivity C-reactive protein, *RBANS* Repeatable Battery for the Assessment of Neuropsychological Status, *DPT* dot-probe task.

*Notes*: ^***^P < 0.001. Correlations were calculated using Spearman’s ρ.

### Relationships among the *BDNF* Val66Met genotype, childhood maltreatment, and attention indices

Numbers of participants with the *BDNF* Val/Val, Val/Met, and Met/Met genotypes were 40 (37.4%), 52 (48.6%), and 15 (14.0%), respectively. The genotype frequency did not deviate from Hardy–Weinberg equilibrium (*χ*^2^(1) = 0.08, *P* = 0.77).

**Fig. 2:**
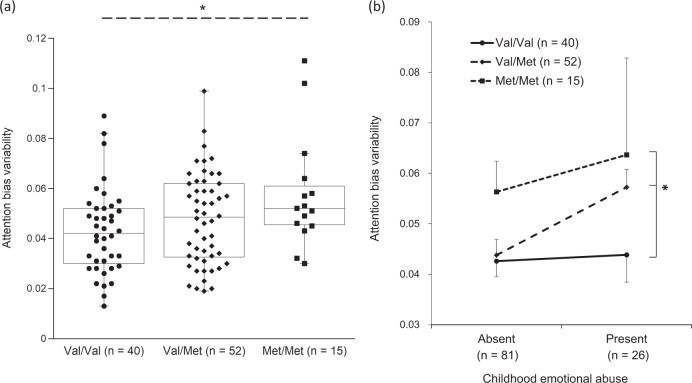
Attention bias variability scores across the *BDNF* Val66Met genotype groups. **a** Combined dot- and boxplot comparing the attention bias variability scores between individuals with the Val/Val (*n* = 40), Val/Met (*n* = 52), and Met/Met (*n* = 15) genotypes. *A significant trend toward greater attention bias variability with increasing numbers of Met alleles (*P* = 0.021 by the Jonckheere–Terpstra trend test). **b** Interaction between the Val66Met genotype and childhood emotional abuse for the attention bias variability scores. Participants were grouped into those with childhood emotional abuse and those without, using the cutoff of 8/9 points for the Childhood Trauma Questionnaire emotional abuse. The solid line with circle markers is the Val/Val genotype, the broken line with diamond markers is the Val/Met genotype, and the dotted line with square markers is the Met/Met genotype. Error bars indicate SEM. *A significant genotype-by-maltreatment interaction (*P* = 0.022 by the two-way ANOVA).

There was no significant association between the Val66Met genotype and attention bias according to the Jonckheere–Terpstra trend test (JT = 1,438.0, *P* = 0.084). However, the trend test showed a significant tendency toward greater ABV with increasing numbers of Met alleles (JT = 2,118.5, *P* = 0.021; Fig. 2a).

Based on the significant association of ABV with the Val66Met genotype and with childhood emotional abuse, we further examined the genotype-by-abuse interaction effect on ABV, using the two-way ANOVA. To this end, the participants were grouped into those with childhood emotional abuse (*n* = 26) and those without (*n* = 81), using the well-defined cutoff of 8/9 points for CTQ emotional abuse^67^. The ANOVA revealed that the genotype-by-abuse interaction was significant (*F* = 2.77, *P* = 0.022); individuals with the Val/Met genotype and those with the Met/Met genotype exhibited even greater ABV when childhood emotional abuse was present whereas those with the Val/Val genotype did not show such an interaction with the abuse (Fig. 2b).

### Exploratory mediation analysis for the role of depressive symptoms in the relationship between childhood emotional abuse and ABV

As described earlier, ABV was significantly positively correlated with depressive symptoms as well as with childhood emotional abuse. In addition, the correlation between CTQ emotional abuse and BDI-II total scores was also significant (rho = 0.216, *P* = 0.014). Based on these tripartite correlations, we further used mediation analysis to investigate the relationship between childhood emotional abuse, ABV, and depression.

The independent variable (X) in this mediation model was childhood emotional abuse, given the temporal precedence. The dependent variable (Y) and moderator variable (M) can be either of the remaining two variables: ABV and depression. Since our primary aim here was to examine the mediation effect of depressive symptoms, we first tested the model (“Model 1”) where (Y) was ABV and (M) was depressive symptoms. In addition, we tested another model (“Model 2”) where (Y) was depressive symptoms and (M) was ABV, considering that this model is also theoretically and temporarily possible. To accommodate this mediation analysis, the raw CTQ emotional abuse score and BDI-II total score were log-transformed (the log-transformation of the latter was calculated after adding “1” to all scores in order to avoid taking the log of zero).

Model 1 indicated a complete mediation, with significant effects of emotional abuse on depression (estimate: 0.225; SE = 0.084, *P* = 0.007) and of depression on ABV (estimate: 0.217; SE = 0.085, *P* = 0.010), and a nonsignificant effect of emotional abuse on ABV (estimate: 0.158; SE = 0.086, *P* = 0.066). The indirect effect was at a trend level (estimate: 0.049; SE = 0.027, *P* = 0.066). Model 2 indicated a partial mediation, with significant effects of emotional abuse on ABV (estimate: 0.207; SE = 0.085, *P* = 0.015), ABV on depression (estimate: 0.216; SE = 0.084, *P* = 0.010), and emotional abuse on depression (estimate: 0.181; SE = 0.085, *P* = 0.033). The indirect effect was at a trend level (estimate: 0.045; SE = 0.025, *P* = 0.079). These results lend some support to the former model in which depression mediates the relationship between childhood emotional abuse and adulthood ABV.

## Discussion

The main findings can be summarized as follows. In healthy adult women, a history of childhood emotional abuse was significantly associated with ABV whereas none of the maltreatment types was associated with attention bias. Proinflammatory activity as indicated by serum hsTNF-α levels was significantly associated with greater ABV. The *BDNF* Val66Met polymorphism was associated with ABV in the manner that ABV significantly increased with increasing numbers of the Met allele. We further observed a significant genotype-by-emotional abuse interaction for ABV, such that Met allele carriers with childhood emotional abuse exhibited even greater ABV while this interactive effect was absent in Val/Val homozygotes.

This is the first study, to our knowledge, to examine the association between childhood maltreatment and ABV in a nonclinical population. Our finding of the significant association between childhood emotional abuse and greater ABV is in line with the evidence for PTSD patients^20,21,25,28,29^. On the other hand, there was no association between childhood maltreatment and attention bias. This seems to be plausible, considering that previous findings of attention bias in trauma-related conditions have been mixed such that attention bias toward threat^9,13,14,19,79^, away from threat^16–18,22–24^, and no bias^15,20,21^ have all been reported. In this study of childhood maltreatment, the absence of association for attention bias, together with the significant relationship between emotional abuse and ABV, suggests that the fluctuation in attention bias towards and away from emotional stimuli over time, rather than constant bias in only one direction, better reflects the emotional attention dysfunction associated with childhood (emotional) trauma. In addition, the observed trend-level correlation between ABV and attention ability suggests that ABV can be partially, albeit not totally, accounted for by general attention ability; this result may suggest both validity and uniqueness of this index.

Although not included in our hypothesis, the tripartite correlations between childhood emotional abuse, ABV, and depression led us to further investigate this association using mediation analysis. The result indicated that depressive symptoms could to some extent mediate the relationship between childhood emotional abuse and ABV. This mediation is plausible, considering that childhood abuse increases the risk of depression^2,4^ and that depression is associated with biased attention to emotional information^36^. In line with our finding, attention control training, a treatment shown to improve ABV in PTSD patients^28,80^, has been found to ameliorate not only PTSD symptoms but also depressive symptoms^28,80,81^. Taken together, it is conceivable that depression can be partly involved in the trauma-related ABV and its treatment mechanism.

Biological factors may underlie the association between childhood trauma and persistent dysregulation in emotional attention. An increasing body of research has demonstrated that childhood maltreatment leaves a lasting impact on stress-related biological systems including the hypothalamic–pituitary–adrenal axis and the inflammatory system^82^. It is proposed that the early trauma-associated alterations in these systems can increase the vulnerability to various psychiatric disorders such as depression and PTSD, as these disorders are also associated with dysregulated glucocorticoid signaling and increased proinflammatory activities^83,84^. In this study, we, therefore, examined the three proinflammatory markers in relation to childhood maltreatment and attention bias indices. The results indicated that increased peripheral inflammation as indexed by serum hsTNF-α levels was significantly associated with greater ABV; however, hsTNF-α levels (and the other inflammatory markers) were not significantly correlated with childhood maltreatment. Although the latter result was not what we hypothesized, these results together suggest that TNF-α may be involved in ABV, possibly independently of childhood trauma. It may be that some other psychosocial factors than childhood maltreatment predominantly contributed to the variation in hsTNF-α levels, given that our nonclinical individuals had overall low levels of childhood maltreatment. Although we can only speculate on the reason for the specific association of ABV with hsTNF-α but not with hsCRP or IL-6, this might be related to the fact that TNF-α is a cytokine while CRP is an acute-phase protein. More specifically, studies show that proinflammatory cytokines such as TNF-α and interleukins can have detrimental effects on cognitive function^85^ and that circulating cytokines in the periphery can affect the brain such as through the blood–brain barrier^86^; while the effects of acute-phase proteins on the brain are less clear. IL-6 is also a cytokine, but it can have anti-inflammatory properties as well as proinflammatory ones^87^, which might explain the absence of a clear association with ABV.

Our results on the *BDNF* Val66Met polymorphism suggest that the Met allele dose-dependently increases ABV and that this effect can at least partly be accounted for by its interaction with childhood emotional abuse. These findings accord with the evidence that the Met allele relates to attention bias^56,57^ and that this SNP interacts with childhood trauma to affect cognitive bias and brain morphology^54,88^. Supporting this, a functional neuroimaging study demonstrates that this SNP predicts amygdala and anterior hippocampus responses to emotional faces in anxious and depressed adolescents^89^. From a broader perspective, the present finding adds to the growing literature on gene–environment interaction for the development of psychopathology. It may also be worth noting that the minor Met allele frequency in our sample was 0.38, which is similar to the frequency of 0.41 reported in a representative genome variation database of Japanese individuals^90^. In contrast, the frequency of this allele is reported to be ~0.15–0.20 among many other populations such as Europeans, according to the Genome Aggregation Database (gnomAD). This relatively higher Met allele frequency in the present sample enabled us to examine its dose-dependent effect, without combining the Val/Met and Met/Met genotypes into a single group. The present analysis where all the three genotype groups were distinguished also accords with the evidence from animal studies demonstrating a dosage effect of the Met allele^91^. On the other hand, our finding warrants further investigations in other ethnic groups since there may be some ethnicity-specific effects of the Met allele^91^.

Several limitations need to be considered when interpreting our findings. First, as the cognitive assessment and inflammatory measurement were derived from cross-sectional data at a single time point, their temporal relationships or long-term trajectories remain speculative. Second, as we only included female participants, it is unknown whether the present findings might be specific to women or common to both sexes. The main reason for the focus on female patients was that this study built on our previous studies of childhood maltreatment, cognitive function, memory bias, inflammation, and the *BDNF* Val66Met polymorphism in women with PTSD^61,92–94^. In addition, it was necessary to consider potential sex differences in this study, given the evidence for differential psychobiological impacts of childhood maltreatment between sexes^95^ and for sexually dimorphic effects of the *BDNF* Val66Met polymorphism^49^. Third, while we calculated ABV by using the bins of trial, some recent studies have used the moving average method to calculate it^25,29^. Although the latter method may be more sensitive in assessing ABV than the former, both calculation methods measure the same concept (i.e., ABV). In addition, the fact that a significant association was found between childhood emotional abuse and ABV in the present study suggests that ABV obtained by using bins is a sufficiently sensitive index that reflects the effect of childhood maltreatment. Still, it is possible that future studies could benefit from adopting the moving average method to calculate ABV. Fourth, we used a retrospective measure (i.e., the CTQ) to assess childhood maltreatment, which might have biased the results. Finally, this study targeted nonclinical individuals, and therefore its clinical significance remains relatively unclear. Relatedly, the exclusion of participants with psychiatric symptoms may have potentially restricted the range of the association between childhood maltreatment and attention bias/ABV. Indeed, the vast majority of our participants did not report any childhood experience of physical abuse or sexual abuse, suggesting that the nonsignificant results for physical/sexual abuse may be attributable to the very small number of individuals with a history of these two types of maltreatment; while this relatively low frequency of childhood physical/sexual abuse (compared to the other types of maltreatment) among the general population is in agreement with previous reports from Japan in which CTQ was used^96,97^. The low maltreatment frequency may have contributed to the lower internal consistency values (i.e., between 0.5 and 0.6) for physical/sexual abuse in our sample as compared to the high values (i.e., between 0.8 and 0.9) for emotional abuse/neglect. The internal consistency value for physical neglect was even lower, which would be attributable not only to the low frequency of this type of maltreatment in our sample but also to the generally lower internal consistency value of this subscale relative to the other four CTQ subscales as reported in previous studies targeting various populations^98,99^. Still, however, it is also possible that childhood emotional abuse more markedly influences ABV than do other types of maltreatment, considering that ABV reflects dysfunctional emotional attention processing. Meanwhile, the frequency of emotional neglect was relatively high in our sample, but this maltreatment type was not significantly associated with ABV. Although the reason for these discrepant results between emotional abuse and emotional neglect for ABV is not clear, the former might have a greater (or more direct) influence on this attentional function than the latter.

In summary, this study shows that childhood emotional abuse is associated with greater ABV in healthy adult women, suggesting that the early-life adversity can have a long-term negative impact on emotional attention control. In terms of biological mechanisms, our findings indicate that increased inflammation as indexed by the elevated blood hsTNF-α level may be involved in ABV, possibly independently of childhood maltreatment. Findings further suggest that the relationship between childhood emotional abuse and ABV could be moderated by the *BDNF* Val66Met polymorphism in the manner that increasing numbers of Met alleles lead to greater ABV by interacting with the emotional abuse. Future studies that examine the association between early-life adversity and ABV among clinical populations, as well as those that investigate the underlying mechanisms, are needed. Efforts will also need to be directed at developing treatments/interventions that target ABV for subclinical and psychiatrically ill individuals.

## Supplementary information

Supplementary methods

Supplementary Figure 1
