# Supplementary material for: Childhood maltreatment history and attention bias variability in healthy adult women: role of inflammation and the BDNF Val66Met genotype
Source: Transl Psychiatry. 2021 Feb 11;11:122. doi: 10.1038/s41398-021-01247-4 (PMC7878504; doi:10.1038/s41398-021-01247-4)
Supplement: Supplementary file 1 — Supplementary methods [file 41398_2021_1247_MOESM1_ESM.docx]

**Supplementary methods**

**Questionnaires**

***The Posttraumatic Diagnostic Scale (PDS)***^1^

We have previously demonstrated a high concordance rate of PTSD diagnosis (95.1%, κ = 0.90)^2^ between the PDS and the Clinician-Administered PTSD Scale^3^, a gold-standard structured interview for the diagnosis of PTSD.

***The Childhood Trauma Questionnaire (CTQ)***^4^

The CTQ has demonstrated adequate psychometric properties as demonstrated by a good fit of the 5-factor structure^4,5^, internal consistency^4,6^, and test-retest reliability^7^. We used the CTQ after translating it from the original English version into Japanese by the first author, which was then back-translated into English by another Japanese researcher, and the back-translated English version was sent to and approved by the original author (Professor David Bernstein).

**Cognitive measures**

***Attention ability and global cognitive function***

The Repeatable Battery for the Assessment of Neuropsychological Status (RBANS)^8,9^ was administered to all participants. The RBANS is a well-established neuropsychological test battery that has demonstrated good psychometric properties among nonclinical and clinical populations^8,10,11^. The RBANS has 12 different subtests and can assess immediate memory, visuospatial construction, language, attention, and delayed memory, as well as their total score indicating global cognition. Scoring was done in accordance with the manual guidelines^8,9^.

**Measurement of proinflammatory markers**

The detection limit for hsTNF-α was 0.6 pg/ml for 65 individuals and 0.15 pg/ml for 53 individuals; this difference was because more sensitive reagent for hsTNF-α became available in the middle of this study. hsTNF-α levels of 11 participants (9.3%) were below the detection limit. The intra- and inter-assay coefficients of variation for hsTNF-α at 0.73 and 6.3 pg/ml ranged between 2.9 and 6.0%. As for IL-6, the detection limit was 0.3 pg/ml; only one subject showed an IL-6 level below this limit. The intra- and inter-assay coefficients of variation for IL-6 at 59.3, 170.8, and 523.0 pg/ml were all less than 2.6%. For hsCRP, the detection limit was 51 ng/ml; hsCRP levels of 7 subjects (5.9 %) were below this limit. The intra- and inter-assay coefficients of variation for hsCRP at 356.0 and 2071.4 ng/ml were all less than 2.1%. Values under the detection limits were treated as 0 (pg/ml or ng/ml).

**References**

1. Foa, E.B. *Posttraumatic Diagnostic Scale (PDS) manual.* (National Computer Systems, Minneapolis, 1995).
2. Itoh, M. et al. The Japanese version of the Posttraumatic Diagnostic Scale: Validity in participants with and without traumatic experiences. *Asian J. Psychiatr.* **25**, 1-5 (2017).
3. Blake, D.D. et al. The development of a Clinician-Administered PTSD Scale. *J. Trauma. Stress* **8**, 75-90 (1995).
4. Bernstein, D.P. et al. Development and validation of a brief screening version of the Childhood Trauma Questionnaire. *Child Abuse Negl.* **27**, 169–190 (2003).
5. Scher, C.D., Stein, M.B., Asmundson, G.J., McCreary, D.R. & Forde, D.R. The childhood trauma questionnaire in a community sample: psychometric properties and normative data. *J. Trauma. Stress* **14**, 843–857 (2001).
6. Gerdner, A. & Allgulander, C. Psychometric properties of the Swedish version of the Childhood Trauma Questionnaire-Short Form (CTQ-SF). *Nord. J. Psychiatry* **63**, 160–170 (2009).
7. Bernstein, D.P. & Fink, L. *Childhood Trauma Questionnaire: A retrospective self-report manual.* (Psychological Corporation, San Antonio, 1998).
8. Matsui, M., Kasai, Y. & Nagasaki, M. Reliability and validity of the Japanese version of the Repeatable Battery for the Assessment of Neuropsychological Status [in Japanese]. *Toyama Med. J.* **21**, 31–36 (2010).
9. Randolph, C., Tierney, M.C., Mohr, E. & Chase, T.N. The Repeatable Battery for the Assessment of Neuropsychological Status (RBANS): preliminary clinical validity. *J. Clin. Exp. Neuropsychol.* **20**, 310–319 (1998).
10. Duff, K. et al. Test-retest stability and practice effects of the RBANS in a community dwelling elderly sample. *J. Clin. Exp. Neuropsychol.* **27**, 565–575 (2005).
11. McKay, C., Casey, J.E., Wertheimer, J. & Fichtenberg, N.L. Reliability and validity of the RBANS in a traumatic brain injured sample. *Arch. Clin. Neuropsychol.* **22**, 91–98 (2007).
