# Supplementary material for: Childhood maltreatment history and attention bias variability in healthy adult women: role of inflammation and the BDNF Val66Met genotype
Source: Transl Psychiatry. 2021 Feb 11;11:122. doi: 10.1038/s41398-021-01247-4 (PMC7878504; doi:10.1038/s41398-021-01247-4)
Supplement: Supplementary file 2 — Supplementary Figure 1 [file 41398_2021_1247_MOESM2_ESM.pptx]

## Slide 1
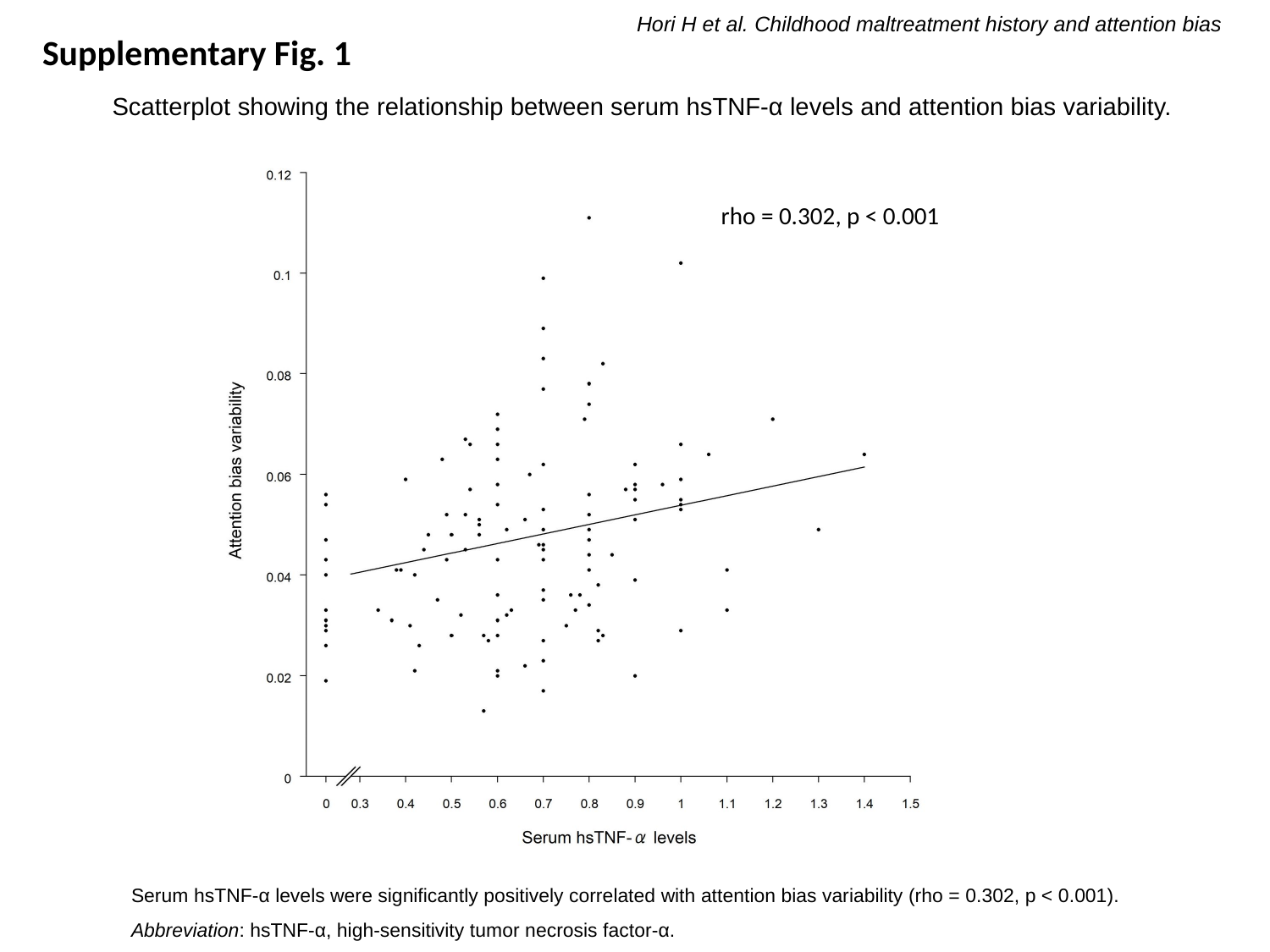

Hori H et al. Childhood maltreatment history and attention bias
Supplementary Fig. 1
Scatterplot showing the relationship between serum hsTNF-α levels and attention bias variability.
rho = 0.302, p < 0.001
Serum hsTNF-α levels were significantly positively correlated with attention bias variability (rho = 0.302, p < 0.001). Abbreviation: hsTNF-α, high-sensitivity tumor necrosis factor-α.
